# Changes of the gut microbiota composition and short chain fatty acid in patients with atrial fibrillation

**DOI:** 10.7717/peerj.16228

**Published:** 2023-12-07

**Authors:** Lingzhi Chen, Jinxin Chen, Yuheng Huang, Yanran Wu, Junfeng Li, Weicheng Ni, Yucheng Lu, Zhenzhen Li, Chuhuan Zhao, Shuting Kong, Hao Zhou, Xiang Qu

**Affiliations:** 1 Wenzhou Central Hospital, Wenzhou, China; 2 The First Affiliated Hospital of Wenzhou Medical University, Wenzhou, China

**Keywords:** Gut microbiota, Atrial fibrillation, Short chain fatty acid

## Abstract

**Background:**

With the establishment of the cardiac-gut axis concept, increasing evidence has suggested the involvement and important regulatory role of the gut microbiota (GM) and short chain fatty acid (SCFA) in cardiovascular diseases. However, the relationship between GM and atrial fibrillation (AF) is still poorly understood.

**Objectives:**

The aim of this study was to investigate whether there were differences in GM and SCFA between AF patients and healthy controls.

**Methods:**

In this study, we enrolled 30 hospitalized patients with AF and 30 matched patients with sinus rhythm (SR). GM species in fecal samples were evaluated through amplicon sequencing targeting the 16Sribosomal RNA gene. The feces SCFAs were describe step by step the quantitative analysis using gas chromatography-mass spectrometry (GC-MS). GM species richness, diversity, differential abundance of individual taxa between AF and SR were analyzed.

**Results:**

AF patients showed decreased species richness and α-diversity compared to SR patients, but there was no statistical difference. The phylogenetic diversity was significant decreased in AF group. The β-diversity indexes revealed significant differences in GM community structure between the AF group and the SR group. After investigated the individual taxa, AF group showed altered relative abundance in several taxa compared to the SR group. linear discriminant analysis (LDA) effect size (LEfSe) analysis revealed, a significant decrease in Bifidobacterium and a greater abundance of Lactobacillus, *Fusobacterium*, *Haemophilus* in AF group compared with the SR group. The abundance of *haemophilus* was negative correlated with isovaleric acid and isobutyric acid.

**Conclusions:**

In AF patients, the GM phylogenetic diversity and β-diversity decreased, the relative abundance altered in several taxa and the bacterial community structure changed as well as the SCFA level. GM and SCFA dysbiosis might play a crucial part in the occurrence and development of AF.

## Introduction

Atrial fibrillation (AF) is the most common arrhythmia in clinical practice, and is associated with high morbidity, mortality, and high medical costs. In addition, with the aging of the population and the increasing number of other complications such as hypertension, diabetes, heart failure, obesity and chronic kidney disease, the incidence of AF is increasing (Hindricks et al., 2020; Go et al., 2001). These complications are reportedly associated with gut microbial dysbiosis (Karlsson et al., 2013; Li et al., 2017). Currently, the pathophysiological mechanism of the occurrence and development of AF mainly focuses on electrical remodeling and atrial muscle remodeling (Hoffmann et al., 2002), but the occurrence and maintenance of AF by gut microbiota (GM) and their product short chain fatty acid (SCFA) have not been discussed in detail, which also hinders the development of targeted therapy.

Recently, GM has been considered as the human second gene pool (Wilson et al., 2017) and, with the establishment of the cardiac-gut axis concept (Ma & Li, 2018), increasing evidences have suggested the involvement and important regulatory role of the GM in cardiovascular diseases such as hypertension (Li et al., 2017; Zuo et al., 2019c), coronary heart disease (Jie et al., 2017) and heart failure (Ahmad, Ward & Dwivedi, 2019). As the deepening understanding of the relationship between GM and cardiovascular disease, experts have found that imbalance of GM can lead to obesity, hypertension and type 2 diabetes through alterations in the immune system and metabolism (Schiattarella et al., 2019; Yoo et al., 2020). These traditional cardiac risk factors play an important role in atrial remodeling of AF (Du, Dong & Ma, 2017). SCFA acts as important molecular signals between the microbiota and host or as metabolic substrates regulating host cellular metabolism and also plays a central role in the diet-gut microbiome-host metabolism axis (Morrison & Preston, 2016). SCFA mainly consist of acetic acid, propionic acid, butyrate acid, isobutyric acid, valeric acid, isovaleric acid and caproic acid. However, there is still a lack of evidence showing whether the alterations of GM and SCFA will affect atrial electrical activity, leading to a similar arrhythmia-inducing effect.

Based on the above effects of GM and SCFA on cardiovascular risk factors, we hypothesized that there was a correlation between GM and SCFA and AF. Therefore, we sought to discover direct evidence of GM and SCFA alterations in AF patients and performed 16S rRNA sequence analysis and used gas chromatography-mass spectrometry (GC-MS) on stool samples from AF patients to investigate the composition and function changes in GM and SCFA. This study is the basis for further research to uncover a causal relationship between GM and SCFA and AF, also explore the preventive measures of delaying the progression of AF.

## Methods

### Study population

This study enrolled 60 patients who hospitalized in the department of cardiology the First Affiliated Hospital of Wenzhou Medical University from January 2020 to December 2020, including 30 patients in the sinus rhythm (SR) group and 30 patients in the AF group. The study protocol was approved by the Ethics Committee in Clinical Research of the First Affiliated Hospital of Wenzhou Medical University, and all study participants provided written informed consent. The approval reference number was clinical research ethics approval number #174. Portions of this text were previously published as part of a thesis (Qu et al., 2019; Qu et al., 2021).

Patients with SR and AF were grouped as previously described by Qu et al. (2019). Specifically, patients were included in the SR group if their electrocardiogram (ECG) suggested sinus rhythm. In addition, SR patients had not received any previous antiarrhythmic treatment, had no history of AF, and had no evidence of AF in the results of electrocardiogram, dynamic electrocardiogram, or ECG telemetry during hospitalization. Patients were categorized to AF based on previous medical history and ECG, dynamic ECG or cardiac telemetry system results during hospitalization. AF was defined as cardiac arrhythmia with “absolutely” irregular RR intervals and no distinct P waves on ECG. The main exclusion criteria were patients with other types of arrhythmias eventually determined by ECG or electrophysiological examination; acute and chronic heart failure patients with New York Heart Association level III–IV requiring hemodialysis or ventilator therapy; liver function incomplete with transaminase was more than upper limit three times; acute or chronic renal insufficiency with creatinine more than three times of the upper limit; patients with acute myocardial infarction with Killip III-IV requiring ventilator support or intra-aortic balloon dilation; patients with hemodynamic instability due to various reasons requiring rescue; patients within one month prior to the enrolled had intestinal diseases or those who had taken probiotics or antibiotics, because these factors may affect GM and SCFA in faeces; patients did not consent to participate in this study or any other reasons that the investigator felt that the patient’s condition would interfere the clinical study. SR and AF’s ECG were blindly adjudicated by a validation committee who referred to the Guidelines for the Management of Atrial Fibrillation (ESC) (Kirchhof et al., 2016).

### Related baseline and clinical samples

The data were collected from the hospital database using a web-based electronic medical record system. Data were collected as previously described by Qu et al. (2021). Specifically, the following demographic and clinical characteristics were extracted: age, gender and body mass index (BMI, kg/m^2^). Traditional cardiovascular risk factors including smoking, alcohol intake, hypertension, diabetes and coronary heart disease. key laboratory data considered as prognostic markers were also collected. Including C-reactive protein (CRP), eGFR (calculated based on serum creatinine), alanine aminotransferase (ALT), aspartate aminotransferase (AST), low-density lipoprotein cholesterol (LDL-C), brain natriuretic peptide (BNP). Baseline peripheral vein blood samples were collected on the morning following hospitalization from patients in stable condition. All the biochemical measurements were performed at the hospital’s laboratory.

### Stool samples collection

Stool samples were collected from middle section of the fresh feces for the first time after hospitalization. Stool samples were divided into well-sealed 250 mg sterilized tubes by the patients themselves, according to our instructions, and stored the samples at −20 °C. Researchers collected the stool samples within 24 h, frozen with liquid nitrogen for 15 min, then stored at −80 °C until analysis.

### DNA extraction and PCR amplification

DNA was extracted from the stool samples and 16S rRNA amplicon sequencing were performed using the NovaSeq PE250 sequencing platform, according to the manufacturer’s instructions. Data were collected as previously described in Chen et al. (2020). Specifically, Genomic DNA of the samples were extracted by SDS-CTAB method and the purity and concentration of DNA were detected by agarose gel electrophoresis. Appropriate amount of DNA was taken and diluted to 1ng/µl in sterile water. Using diluted genomic DNA as a template, the V4 region of the bacterial 16S rRNA was amplified by PCR with the specific primers 515F(5′-GTGCCAGCMGCCGCGGTAA-3′) and 806R(5′-GGACTACHVGGGTWTCTAAT-3′) labeled in a 12 bp barcode. Using high-fidelity PCR Master Mix with GC Buffer (Phusion^®^; New England Biolabs, Ipswich, MA, USA) and high-fidelity enzyme to ensure amplification efficiency and accuracy.

### Sequencing and data processing

Reads for each sample were demultiplex from the raw data according to Barcode sequence and PCR primer sequence. Original tags data (raw tags) were formed by splicing the reads from each sample using FLASH (V1.2.7, http://ccb.jhu.edu/software/FLASH/) (Magoč & Salzberg, 2011). The spliced raw tags were strict filtered (Bokulich et al., 2013) to get high-quality clean tags according to published protocols (Qiime V1.9.1, http://qiime.org/scripts/split_libraries_fastq.html) (Caporaso et al., 2010). Clean tags detected chimeric sequences by comparing with the species annotation database (https://github.com/torognes/vsearch/) (Rognes et al., 2016), and finally removed the chimeric sequences to obtain the final effective tags. Operational Taxonomic Units (OTUs) were clustered against the Uparse software (Uparse V7.0.1001, http://www.drive5.com/uparse/) (Haas et al., 2011) at 97% identity for all effective tags. The representative sequence of the OTUs was analyzed by mothur method and SILVA138 (http://www.arb-silva.de/) (Edgar, 2013) SSU rRNA database (Wang et al., 2007). MUSCLE program (Version 3.8.31, http://www.drive5.com/muscle/) (Quast et al., 2013) was used for fast multi-sequence alignment to obtain the phylogenetic relationships of all OTUs. Finally, the data of all samples were homogenized, and the sample with the least amount of data was taken as the standard for homogenization. The subsequent Alpha and Beta diversity analysis were based on the homogenized data.

### Alpha diversity analysis

We analyzed within-community microbial diversity by using alpha diversity analysis. Qiime v1.9.1 was used to calculate Observed-species, Chao1, ACE, Shannon, Simpson, PD_whole_tree indexes. R version 2.15.3 (R Core Team, 2013) was used to plotted the rarefaction curve, rank abundance curve and species accumulation boxplot. The difference analysis between AF group and SR group of alpha diversity indexes were conducted by *t*-test and Wilcox test.

### Beta diversity analysis

We analyzed the dissimilarities in the microbiomes by using beta diversity analysis. Qiime v1.9.1 was used to calculate unweighted unique fraction metric (Unifrac) between AF group and SR group. Principal co-ordinates analysis (PCoA) and non-metric multi-dimensional Scaling (NMDS) diagrams were drawn using R v2.15.3. We performed PCoA by using WGCNA, STATS and GGplot2 packages of R and performed NMDS analysis by using vegan package of R. Analysis of similarities (Anosim), multi-response permutation procedures (MRPP) and Adonis (permutational multivariate analysis of variance) were analyzed by using the Anosim function, MRPP function and Adonis function of R vegan package respectively. The difference analysis in the unweighted Unifrac between the AF and SR groups were conducted by *t*-test and Wilcox test in R software.

### LEfSe and PICRUSt analysis

LEfSe software was used for the linear discriminant analysis (LDA) effect size (LEfSe) analysis and the threshold of LDA score (log10) was set to 4. Permutation tests were performed between groups at various classification levels (Phylum, Class, Order, Family, Genus and Species) by using the metastats analysis of R software, and *P*-values were obtained. Then, the Benjamini and Hochberg false discovery rate method was used to correct *P*-values and obtained *q*-values (Edgar, 2004). The difference analysis between the AF and SR groups were also using the *t*-test and Wilcox test in R software. GM functional was predicted by using the phylogenetic investigation of communities by reconstruction of unobserved states (PICRUSt) (Langille et al., 2013). The detailed prediction process was referred to http://picrust.github.io/picrust/tutorials. Function prediction based on kyoto encyclopedia of genes and genomes (KEGG) database was performed based on 16S sequencing data. Additionally, Spearman correlation was used to associate abundant differential taxa with SCFA.

### SCFA sample processing and gas chromatography analysis

The GCMS system mainly consisted of gas mass spectrometer (Thermo TRACE 1310-ISQ LT, USA), vortex mixer (QL-866, China) and refrigerated centrifuge (Xiangyi, China). The SCFA were extracted as described previously (Hoving et al., 2018). Briefly, 50 mg of the sample was added with 50 µL15% phosphoric acid, 100 µL of 125 µg/mL internal standard (isohexanoic acid) solution and 400 µL of diethyl ether homogenate for 1 min. The sample was centrifuged at 12,000 RPM for 10 min at 4 °C, and the supernatant was transferred to a chromatographic vial for GC analysis. Chromatographic conditions: agilent HP-INNOWAX capillary column (30m* 0.25 mm ID* 0.25 µm). The injection volume was 1 µl, and the split ratio was 10:1. The carrier gas was helium with a flow rate of 1.0 mL/min.

### Statistical analysis

The statistics for continuous variables were expressed as means and standard deviations or as median and interquartile range when the distribution was not normal. Categorical variables as frequencies and proportions. Baseline characteristics were compared between AF group and SR group. A Fisher’s exact test or chi-square test was used to identify differences in categorical data, and independent-sample *t* test (data with normal distribution) or Mann–Whitney *U* test (data with non-normal distribution) were used to identify differences in quantitative data. Spearman correlation analysis was used to identify the correlation between fecal SCFA and GM. Positive *R*-values represented positive correlation and negative R-values represented negative correlation. Statistical analyses were performed using SPSS software version 26 (SPSS, Inc., Chicago, IL, USA). *P* values <0.05 were considered statistically significant.

The raw sequencing reads of this study have been deposited in National Population Health Data Center of China: https://www.ncmi.cn//phda/dataDetails.do?id=CSTR:17970.11.Z061U.202204.366.V1.0.

## Results

### Baseline characteristics of the study cohort

In the current study, 60 patients consisting of 30 SR patients and 30 AF patients were included in our study. The baseline clinical characteristics of the two groups were shown in Table 1. The most baseline clinical characteristics among the SR and AF groups were similar. Compared to the SR group, AF patients were presented with higher BNP level.

**Table 1:** Baseline clinical characteristics of patients.

Clinical parameters	Sinus rhythm group (*n* = 30)	Atrial fibrillation group (*n* = 30)	*P* value
Demographics			
Age (y)	64.93 ± 10.27	68.20 ± 9.54	0.207
Gender (male N%)	10 (33.3)	17 (56.7)	0.069
Body mass index (kg/m^2^)	24.22 ± 2.82	24.09 ± 2.34	0.855
Laboratory measurements			
CRP (mg/L)	2.59 ± 5.56	2.83 ± 4.41	0.826
ALT (U/L)	25.63 ± 21.77	27.67 ± 22.65	0.724
AST (U/L)	26.73 ± 12.96	26.90 ± 10.21	0.956
eGFR(ml/min/1.73m^2^)	85.66 ± 17.91	80.46 ± 24.32	0.349
LDL-C (mmol/L)	2.33 ± 0.72	2.20 ± 0.66	0.444
BNP (pg/mL)	23.00(10.00–35.00)	150.00(108.00–321.00)	<0.001^*^
Medical history, *n* (%)			
Diabetes mellitus	6(20.0)	9(30.0)	0.371
Hypertension	16(53.3)	17(56.7)	0.795
Smoking	8(26.7)	13(43.3)	0.176
Alcohol use	5(16.7)	9(30.0)	0.222
Coronary heart disease	10(33.3)	10(33.3)	1.000

**Notes.**

Continuous variables are expressed as mean ± standard deviation for normally distributed, and median and interquartile range when the distribution was not normal. Categorical data are shown as frequencies and percentages.

Abbreviations CRP C-reactive protein ALT Alanine aminotransferase AST Aspartate aminotransferase eGFR estimated glomerular filtration rate LDL-C Low-density lipoprotein cholesterol level BNP brain natriuretic peptide

* *P* < 0.05.

### Fecal SCFA levels in SR and AF groups

**Figure 1:**
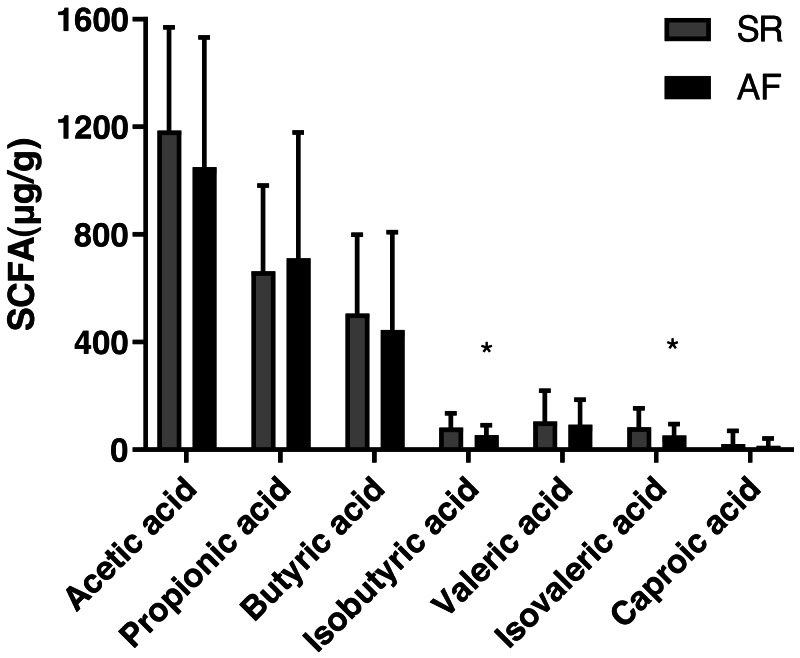
Fecal levels of short chain fatty acids in sinus rhythm and atrial fibrillation patients. Bite: ^∗^*P* < 0.05.

The concentrations of acetic acid, propionic acid, butyrate acid, isobutyric acid, valeric acid, isovaleric acid and caproic acid in feces are shown Table 2 and Fig. 1). The isobutyric acid level was significantly decreased in AF group (53.75 ± 37.49) compared with that in SR group (82.57 ± 52.23, *P* = 0.017). Besides, compared with SR patients (83.56 ± 70.66), the isovaleric acid was significantly decreased in AF patients (52.34 ± 43.50, *P* = 0.044). However, no significant differences were found in levels of acetic acid (*P* = 0.232), propionic acid (*P* = 0.653), butyric acid(*P* = 0.476), valeric acid (*P* = 0.655) and caproic acid (*P* = 0.511) between the two groups.

**Table 2:** Fecal levels of short chain fatty acid for patients with sinus rhythm and atrial fibrillation.

Markers	Sinus rhythm group (*n* = 30)	Atrial fibrillation group (*n* = 30)	*P* value
Acetic acid (µg/g)	1,185.84 ± 384.29	1,049.59 ± 483.22	0.232
Propionic acid (µg/g)	663.93 ± 317.77	710.67 ± 469.00	0.653
Butyric acid (µg/g)	505.47 ± 294.24	444.22 ± 363.79	0.476
Isobutyric acid (µg/g)	82.57 ± 52.23	53.75 ± 37.49	0.017^*^
Valeric acid (µg/g)	104.71 ± 114.05	92.65 ± 92.86	0.655
Isovaleric acid (µg/g)	83.56 ± 70.66	52.34 ± 43.50	0.044^*^
Caproic acid (µg/g)	21.21 ± 49.17	14.42 ± 27.38	0.511

**Notes.**

Data are mean ± SD.

* *P* < 0.05.

### The differences of gut microbiota profiles between SR and AF groups

We drew the rarefaction curves to show that two groups approached a plateau, identifying that the sequencing depth was adequate in this study (Fig. S1A). Then we plotted the rank abundance curves to compare the richness and evenness between SR and AF groups and found reduced richness and evenness in AF patients (Fig. S1B). We further drew the species cumulation boxplot to determine the adequacy of sample size of our research and assess community richness (Fig. S1C). These curves indicated that majority of microbial diversity had been captured.

**Figure 2:**
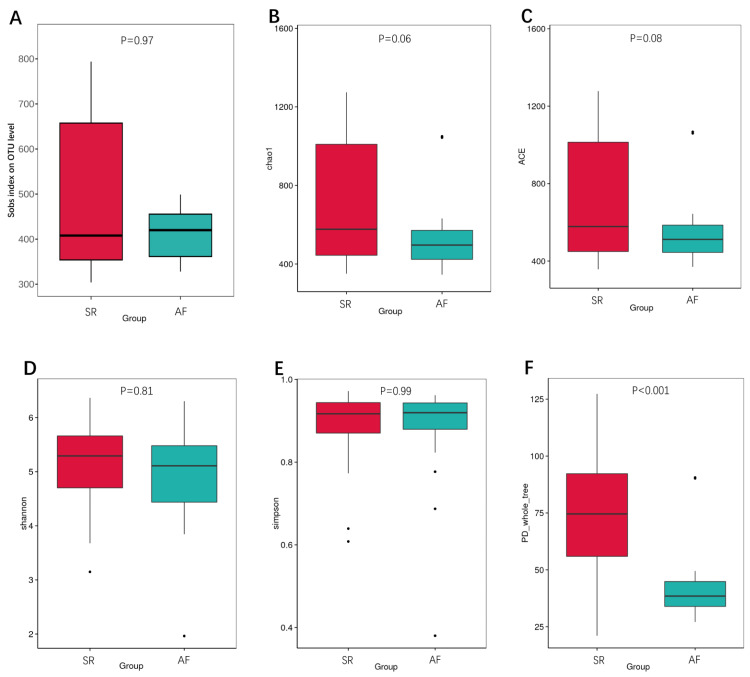
The differences of gut microbiota profiles between SR and AF groups. Changes in the gut microbiota *α*-diversity in atrial fibrillation patients. (A–C) Boxplots for comparison of species richness (observed species, chao1, ACE), (D–E) *α*-diversity (shannon, simpson), (F)and phylogenetic diversity(PD whole tree) between AF patients and SR patients. Data are presented as mean ±standard error of the mean with individual outliers also shown. The data indicated that SR patients had a higher abundance and diversity than in AF patients.

We also calculated the indexes between SR and AF patients to compare the richness by Sobs index, Chao1 and ACE (Figs. 2A, 2B, 2C), *α*-diversity by Shannon and Simpson (Figs. 2D, 2E), and phylogenetic diversity by PD whole tree (Fig. 2F). In the Sobs index, we showed unique OTU with specific relative abundance threshold >0.01% to filter out potential noise from sequencing error. These indexes suggested decreased species richness and *α*-diversity in AF patients compared to SR patients, but there were no statistical difference. However, the phylogenetic diversity by PD whole tree was significant decreased in AF group (*P* < 0.001). It indicated that SR patients had a higher abundance and diversity than in AF patients.

**Figure 3:**
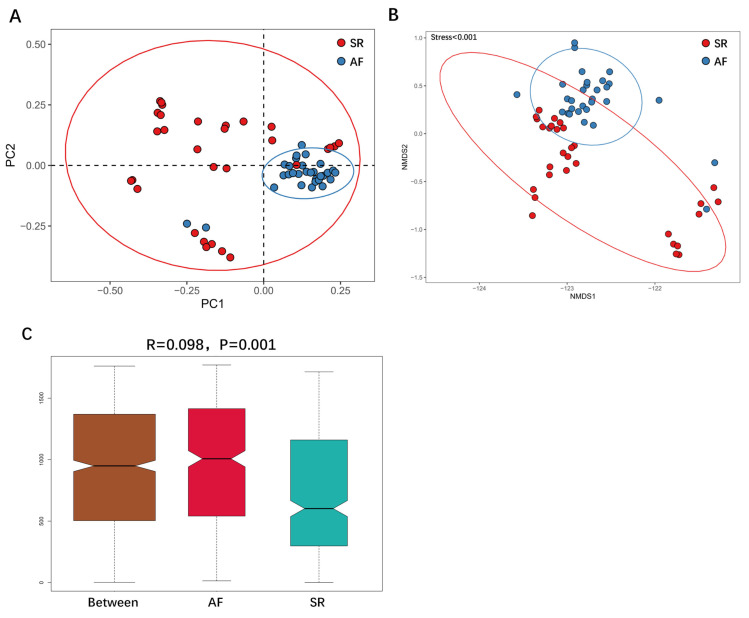
The differences of gut microbiota profiles between SR and AF groups. *β*-diversity analysis using unweighted Unifrac analysis to compare similarities among gut microbial communities. Principal co-ordinates analysis (PCoA) plotted with unweighted Unifrac (A) and Non-metric Multi-Dimensional Scaling (NMDS) plotted with unweighted Unifrac (B) were used to compare the sample distribution between AF patients and SR patients. Anosim analysis (C) was used to identify the similarities of two groups. SR, sinus rhythm; AF, atrial fibrillation.

We then applied unweighted Unifrac analysis to compare similarities among GM communities (*β*-diversity). PCoA and NMDS diagrams revealed more clustered distribution from AF patients compared to SR patients. (Figs. 3A, 3B). Anosim analysis showed that the inter-group difference between AF group and SR group was significantly greater than the intra-group difference, indicating that grouping was meaningful (Fig. 3C. *R* = 0.098, *P* = 0.001). Both analysis suggested reduced *β*-diversity in AF group, which was further supported by MRPP analysis (*A* = 0.010, observed △ = 0.700,expected △ = 0.707, *P* = 0.001) and Adonis analysis (*R*^2^ = 0.036, *P* = 0.001).

**Figure 4:**
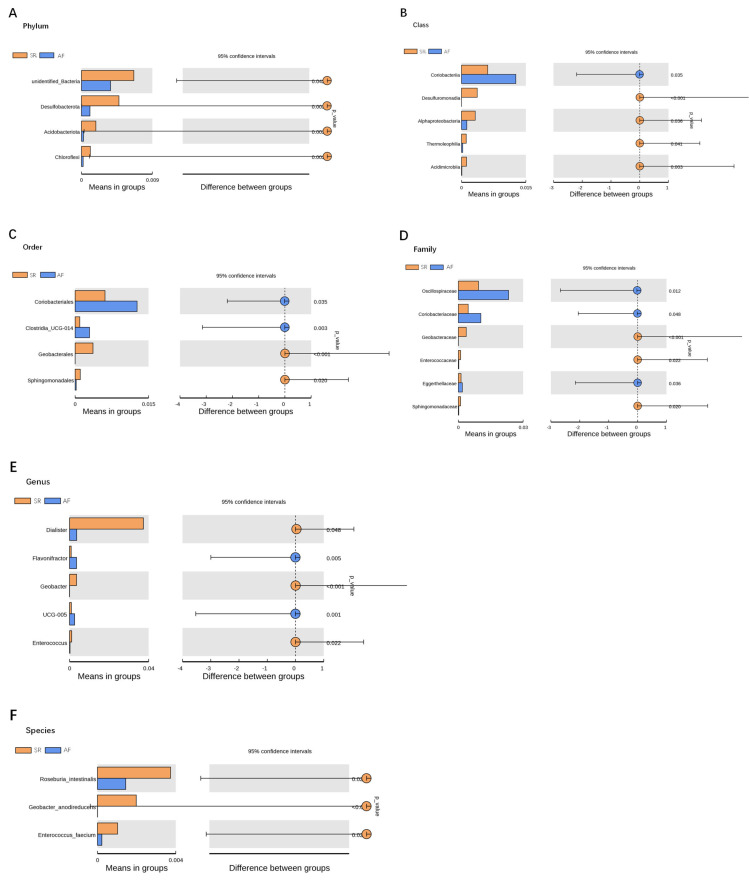
Gut microbiota differences between SR and AF patients detected by *t*-tests. Gut microbiota differences between SR and AF patients detected by *t*-tests. Gut microbiota is compared between AF patients and SR patients at phylum (A), class (B), order (C), family (D), genus (E), and species (F) levels. Only the taxa with statistically significant difference are plotted. The bars on the left side of each figure show the relative abundance. On the right side of each figure, the center of circles represents the difference between the means of the two groups. The error bars represent the 95% confidence interval. *P*-values of unpaired *t*-test are listed on the right (*n* = 30 for AF patients, *n* = 30 for SR patients). SR, sinus rhythm; AF, atrial fibrillation.

**Figure 5:**
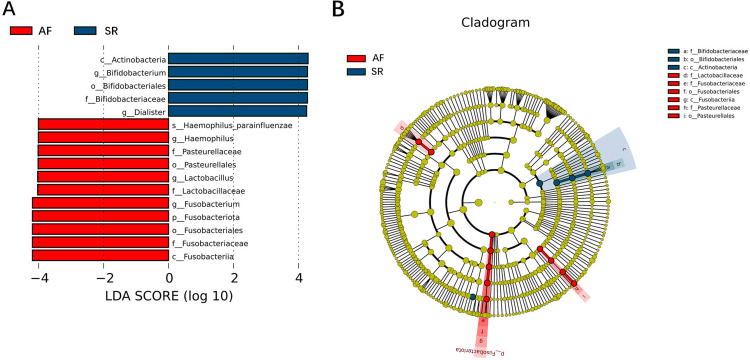
Gut microbiota differences between AF patients and SR patients detected by LEfSe analysis. Gut microbiota differences between AF patients and SR patients detected by LEfSe analysis. (A) The histogram of LDA value distribution showed the species with LDA Score more than the threshold value 4. Species with significant abundance differences in different groups are displayed. The length of the bar chart represents the impact of different species. (B) In a cladistic diagram, circles radiating from inside out represent the level of classification from phylum to species. Each small circle at different classification levels represents a classification at that level, and the diameter of the small circle is proportional to the relative abundance. SR, sinus rhythm; AF, atrial fibrillation; LDA, linear discriminant analysis.

To show the difference in microbiome distribution between AF and SR cohorts for more direct visualization, we made the bar plot to showing the top 10 GM relative abundance of different patients by genus level (Fig. S2). To investigate the differential abundance of individual taxa, we first ran t-tests of the relative abundance of each taxon between SR and AF patients at different phylogenetic ranks, and found significant difference in four phylum, five classes, four orders, six families, five genera, and three species (Fig. 4). To analyze the statistical differences in microbial communities between the SR group and the AF group, we also performed the LEfSe analysis, which coupled statistical significance with biological consistency and effect relevance. The histogram reflected the LDA scores greater than 10^4^ that were computed for the features at the OTU level (Fig. 5A). Upon analyzing the fecal samples, a total of 5 species of bacteria were abundant in the SR group and 11 species were abundant in the AF group. The genera that were enriched in the AF group were *Haemophilus_parainfluenzae, Haemophilus, Pasteurellaceae*, *Pasteurellales*, *Lactobacillus*, *Lactobacillaceae*, *Fusobacterium*, *Fusobacteriota*, *Fusobacteriales*, *Fusobacteriaceae* and *Fusobacteriia*. Several genera were found in significantly high abundances in the SR group. These included *Actinobacteria*, *Bifidobacterium*, *Bifidobacteriales*, *Bifidobacteriaceae* and *Dialister*. Cladograms of the taxa with LDA values > 4.0 were depicted (Fig. 5B). Cladograms were generated from the LEfSe analysis, which showed the most differentially abundant taxa enriched in microbiota with green for the SR group and red for the AF group. Each small circle at different classification levels represented a classification at that level, and the diameter of the small circle was proportional to the relative abundance. The AF group showed a significant decrease in the class *Actinobacteria*, order *Bifidobacteriales* and family *Bifidobacteriaceae* and a greater abundance of the class *Fusobacteriia*, order *Fusobacteriales*, order *Pasteurellales*, family *Lactobacillaceae*, family *Fusobacteriaceae* and family *Pasteurellaceae* when compared with the SR group.

**Figure 6:**
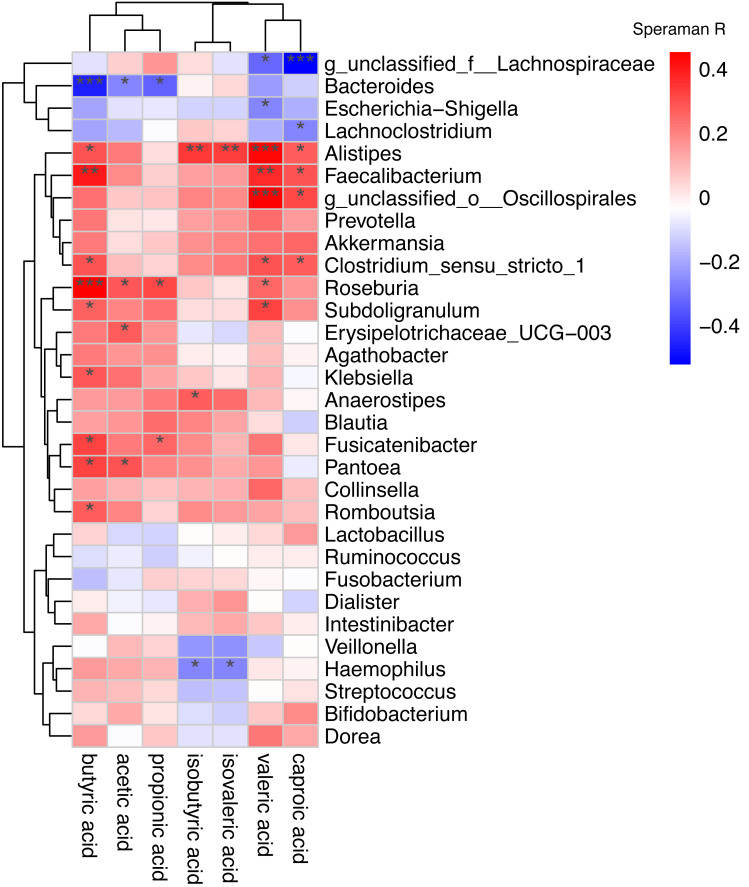
Correlation of the gut microbiota with the levels of fecal short chain fatty acids. Correlation of the gut microbiota with the levels of fecal short chain fatty acids. The heatmap shows the correlation coefficient between bacterial taxa and the level of fecal short chain fatty acids and the genus level. The R values are represented by gradient colors, where red and green cells indicate positive and negative correlations, respectively. ^∗^*P* < 0.05; ^∗∗^*P* < 0.01; ^∗∗∗^*P* < 0.001.

Spearman correlation was used to associate the differentially abundant taxa with the fecal levels of SCFA at the genus level (Fig. 6). *Haemophilus* which significantly increased in AF was negative correlated with isovaleric acid (rs =−0.27, *P* = 0.039) and isobutyric acid (rs =−0.26, *P* = 0.043). Other GM that was significantly increased in the AF and sinus rhythm groups did not differ significantly in SCFA.

Figure 3 was more about inter-group differences and intra-group aggregation, Fig. 3A was diagrammed by PCoA, while Fig. 3B was diagrammed by NMDS. PCoA and NMDS are mainly used to reflect the relation of sample distance matrix. The difference between the two lies in that, PCoA is based on the analysis of similarity distance between samples, while NMDS focuses more on reflecting the ordering relation of values in the distance matrix and weakens the absolute difference degree of values. In Fig. 3B, the smaller the Stress value is, the more complete information in the high-dimensional is retained. Figure 3C was based on the rank of Bray-Curtis distance value to test the significance of inter-group differences, which was used to test whether the inter-group differences were significantly greater than the intra-group differences. Fig. 4 was obtained by a *t*-test using abundance to identify species with significant differences (*P* < 0.05). Figure 5A mainly showed the species with significant difference abundance between the two groups. The histogram of distribution of LDA values showed species with an LDA Score greater than 4, namely the species with statistical difference between groups. Figure 5B was a Cladogram. The circles radiating from inside to outside represent taxonomic levels from phylum to species, which could more directly showed the species with significant difference abundance between the two groups.

### Prediction of functional gene content

To realize the changes of intestinal microbial function in patients with AF, we based on KEGG database used PICRUSt. PICRUSt revealed six biological metabolism pathways at Level 1 pathways including metabolism, genetic information processing, environmental information processing, cellular processes, human diseases and organismal systems. Among them, metabolism, genetic information processing and environmental information processing dominated, accounting for 44.42%–48.17%, 17.29%–20.77% and 13.48%–17.92%, respectively. But, the six pathways in the AF group were not statistically different from those in the SR group. In KEGG pathway hierarchy level 2, we discovered 41 sub-functions. The top 10 were membrane transport, carbohydrate metabolism, amino acid metabolism, replication and repair, energy metabolism, translation, poorly characterized, metabolism of cofactors and vitamins, cellular processes and signaling, nucleotide metabolism. Within the two predicted functional categories at hierarchy level 2 pathway including amino acid metabolism (AF group 9.35% ± 0.33% *vs* SR group 9.58% ± 0.30%; *P* = 0.008) and metabolism of cofactors and vitamins (AF group 4.14% ± 0.13% *vs* SR group 4.25% ± 0.19%; *P* = 0.017) significantly decreased in AF patients. In KEGG pathway hierarchy level 3 we discovered 305 gene pathways. Oxidative phosphorylation(AF group 1.07% ± 0.06% *vs* SR group 1.11% ± 0.07%; *P* = 0.042), glycine, serine and threonine metabolism (AF group 0.97% ± 0.06% *vs* SR group 0.94% ± 0.05%; *P* = 0.024), photosynthesis proteins(AF group 0.79% ± 0.03% *vs* SR group 0.81% ± 0.03%; *P* = 0.047) and novobiocin biosynthesis (AF group 0.41% ± 0.06% *vs* SR group 0.44% ± 0.06%; *P* = 0.033) were significantly increased in the SR group. Whereas, naphthalene degradation(AF group 0.13% ± 0.02% *vs* SR group 0.12% ± 0.02%; *P* = 0.027) was significantly increased in the AF group. In KEGG pathway hierarchy level K we discovered 6309 taxa. K00145(N-acetyl-gamma-glutamyl-phosphate reductase: AF group 0.12% ± 0.02% *vs* SR group 0.13% ± 0.01%; *P* = 0.045) and K06148 (ATP-binding cassette: AF group 0.12% ± 0.02% *vs* SR group 0.11% ± 0.02%; *P* = 0.026) were the highest abundance gene pathways of GM in SR and AF patients, respectively

## Discussion

In this article, we found those patients with AF had higher BNP levels than those with SR. Previous studies have also considered that atrial and ventricular myocyte stretching, counter regulation of myocardial fibrosis and inflammation response, and downregulation of neutral endonuclease in patients with AF are the main causes of elevated BNP levels. Our study had similar result to previous studies (Kerr & Brandon, 2022).

In this study, we discovered that AF patients compared to SR patients had lower species richness and *α*-diversity, but there were no significant differences. Whereas, the phylogenetic diversity of AF patients was significant decreased. Furthermore, the *β*-diversity indexes revealed statistical difference in GM community structure in AF group and SR group. More importantly, *t*-test and LEfSe analysis revealed that several taxa showed significant difference in abundance between AF patients and SR patients. Especially in AF group, a significant decreased in *Bifidobacterium* and a greater abundance of *Lactobacillus*, *Fusobacterium*, *Haemophilus*. In addition, the concentrations of isobutyric acid and isovaleric acids in SCFA decreased significantly in patients with AF and this decrease was inversely correlated with the abundance of *Haemophilus* in patients with AF. In our opinion, the variation of these bacteria and SCFA may be the key of AF development and establishment. Therefore, we hypothesized that the increase or decrease of specific group of GM and specific kinds of SCFA may negatively affect human health, interfere with the progression of AF, and may even play an important role in the establishment of AF. As a consequence, a discriminant model based on bacterial characteristic spectrum could be established, which can be used as a biomarker for AF in the future, and intervention strategies targeting GM can be used to improve the progression of AF.

For all we know, SCFA level and GM abundance as well as diversity have been assessed in variety of diseases, especially in cardiovascular disease such as hypertension (Li et al., 2017; Zuo et al., 2019c), atherosclerosis (Koeth et al., 2013), coronary heart disease (Jie et al., 2017) and heart failure (Ahmad, Ward & Dwivedi, 2019). In these disease states, the diversity, composition and related metabolic functions of GM changed, leading to disruption of important physiological processes, including inflammation, lipid metabolism, bacterial translocation, and glucose homeostasis, which may contribute to the development and progression of cardiovascular disease (Tang et al., 2019). A recent metagenome association study from atherosclerosis patients observed that increased relative abundance of *Escherichia coli*, *Enterobacter aerogenes*, *Klebsiella* and *Streptococcus* were associated with an increased incidence of atherosclerotic cardiovascular disease (Jie et al., 2017). In addition, another study also showed that the GM abundance of heart failure patients decreased, and the composition of GM changed significantly with the progress of the disease, that is, the increase of pathogenic bacteria, including *Campylobacter*, *Shigella*, *Salmonella*, *Yersinia enterocolitica* and *Candida* species (Pasini et al., 2016). SCFA, as metabolites of the GM, were thought to have beneficial effects on cardiovascular diseases through some mechanisms including inhibition of endothelial inflammation, protecting against oxidative stress-induced vascular damage and as microbial signals to maintain host immunity (González-Bosch et al., 2021). Therefore, assessing the abundance and diversity of GM and SCFA concentration in feces can reflect the imbalance of intestinal environment and predict the disease status of the patient, which is characterized by more harmful bacteria, fewer symbiotic or beneficial bacteria and lower SCFA level. This is consistent with our study.

Our study found that AF as a common cardiovascular disease, the richness and diversity of GM and SCFA level also changed. However, it was different from the previous report (Zuo et al., 2019a). It was worth noting that the increased *Lactobacillus*, *Fusobacterium* and *Haemophilus* in AF patients were also reflected in other cardiovascular diseases, such as heart failure (Wang et al., 2021), hypertension (Del Chierico et al., 2021), and coronary heart disease (Liu et al., 2019). For example, one study had found that *Haemophilus* could reduce nitric oxide levels, promote endothelial dysfunction and increase cardiovascular risk (Pignatelli et al., 2020). Some articles have explored the effect of SCFA on AF. Yang et al. found the SCFA related gene synthesis changed in the gut of AF patients (Zhang et al., 2021). Furthermore, SCFA was found to alleviate atrial remodeling in patients with atrial fibrillation through GPR43/NLRP3 signaling pathway (Zuo et al., 2022). Therefore, we have reason to believe that this alteration plays an important role in the development of cardiovascular diseases. Although the underlying mechanisms remain unclear, some cardiovascular diseases share some common pathophysiological pathways including myocardial remodeling, endothelial dysfunction (Incalza et al., 2018) and inflammatory reaction (Donath, Meier & Böni-Schnetzler, 2019). More recently, more and more people also have recognized that AF is also associated with atrial remodeling, systemic inflammation and endothelial dysfunction, rather than just an atrial disease (Wijesurendra & Casadei, 2019; Corban et al., 2021). Repairing defective GM, regulating abnormal SCFA level and re-establishing a fully functioning ecological population may mitigate the progression of cardiovascular disease, including AF. Intervention the intestinal microecosystem and SCFA level toward beneficial changes may provide a new treatment for cardiovascular diseases, such as AF, caused by intestinal microbiome disorders or other potential disorders related to ecological disorders (Zhao et al., 2018).

To date, a number of observational studies have described the changes of GM in AF patients, but these studies have been inconsistent about GM changes (Zuo et al., 2019a; Tabata et al., 2021; Zuo et al., 2020; Zuo et al., 2019b). Zuo et al. founded that the GM of AF patients was different from SR patients including a significant elevation in microbial diversity and a specific change of gut microbiota composition. Increased abundance of *Ruminococcus, Streptococcus*, and *Enteroccus*, while decreased abundance of *Faecalibacterium*, *Alistipes*, *Oscillibacter*, and *Bilophila* were detected in AF patients (Zuo et al., 2019a; Zuo et al., 2020; Zuo et al., 2019b). Another study showed that GM richness was lower in AF patients, reduction of *Enterobacter* and overgrowth of *Parabacteroides*, *Lachnoclostridium*, *Streptococcus*, and *Alistipes* were detected in AF patients (Tabata et al., 2021). Notably, the bacterial variation in our study exhibited some unique features that unlike those in previous reports. Complex and diverse factors involved in pathophysiological processes may be partly responsible for this inconsistent phenomenon, whereas all of the above studies suggested that GM composition was different in AF patients and SR patients. In addition, regulating the composition of the microbiome through probiotics, diet patterns, sleep habits and physical activity, may affect the progression of cardiovascular disease and risk factors associated with cardiovascular disease (Oniszczuk et al., 2021; Yan et al., 2021; St-Onge & Zuraikat, 2019), which is also expected to be a new treatment method for AF and will be explored in our future study.

### Study limitations

There were several limitations in this study. The most importantly, this study was an observational study and could not determine the causal relationship between GM or SCFA and AF; therefore, a prospective cohort study is needed to determine whether changes in GM or SCFA are associated with the etiology of AF. Furthermore, the sample size of this study was small, thus, larger studies are needed to validate our results. In addition, we cannot exclude the influence of confounding factors, such as sleep, diet and drugs, on the GM or SCFA of AF patients.

## Conclusions

This study showed that GM and SCFA significantly differed between AF patients and SR patients. The species of gut bacterial variation and the relationship with SCFA in our study exhibited some unique features compared to previously reported. Extensive researches in animal models and clinical trials even the prospective cohort studies are needed to better define the causal relationship between the alteration of GM or SCFA and the etiology of AF.

## Supplemental Information

10.7717/peerj.16228/supp-1 Supplemental Information 1 Raw data

10.7717/peerj.16228/supp-2 Supplemental Information 2 Absolute OTU

10.7717/peerj.16228/supp-3 Supplemental Information 3 The richness of gut microbiota between SR and AF groups The gut microbiota richness in atrial fibrillation patients. (A) Rarefaction curves to show the adequate depth of the sequencing. (B) Rank abundance curve to show the relative species abundance of each sample from AF and SR patients. (C)Species accumulation boxplot to show that the number of samples is also adequate.

10.7717/peerj.16228/supp-4 Supplemental Information 4 The community composition of gut microbiota at the genus-level (A) Relative abundance of the top ten gut microbiota in each AF and SR patients. (B) Relative abundance of the top ten gut microbiota in AF and SR groups. The figure showed the species with top 10 gut microbiota in relative abundance.

## Additional Information and Declarations

### Data Availability

The following information was supplied regarding data availability: The raw sequencing reads of this study are available in the Supplementary File and at the National Population Health Data Center of China: https://doi.org/10.12213/11.Z061U.202204.366.V1.0.

### Clinical Trial Registration

The following information was supplied regarding Clinical Trial registration: ChiCTR2100050063
