# Supplementary figures and images for: Changes of the gut microbiota composition and short chain fatty acid in patients with atrial fibrillation
Source: PeerJ. 2023 Dec 7;11:e16228. doi: 10.7717/peerj.16228 (PMC10710774; doi:10.7717/peerj.16228)

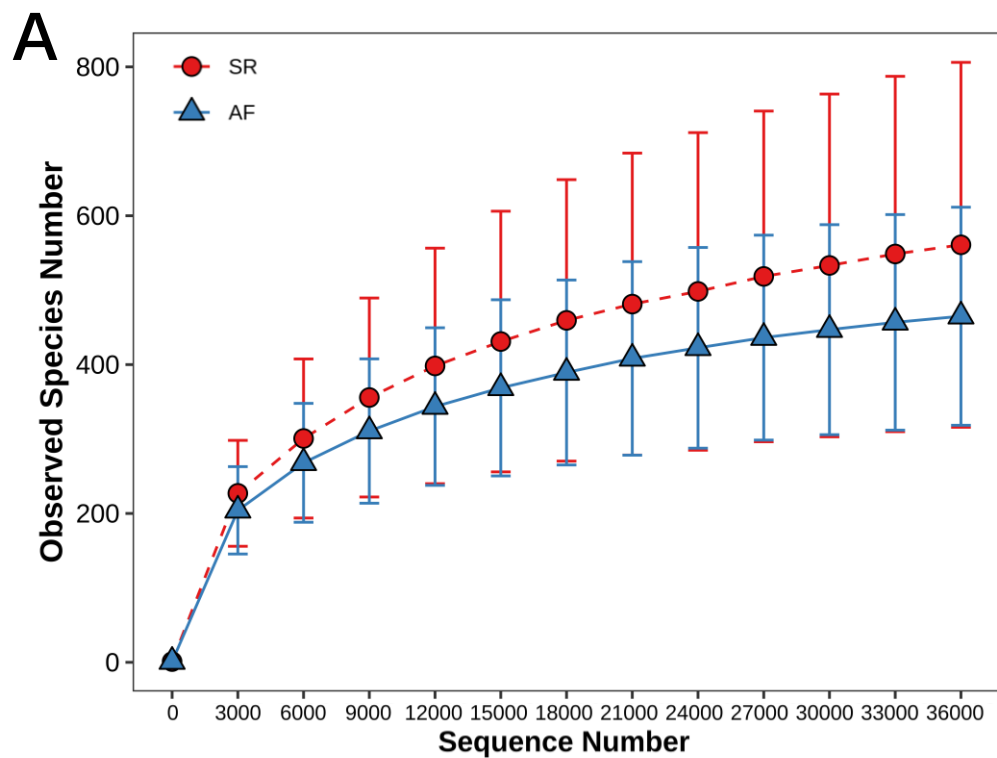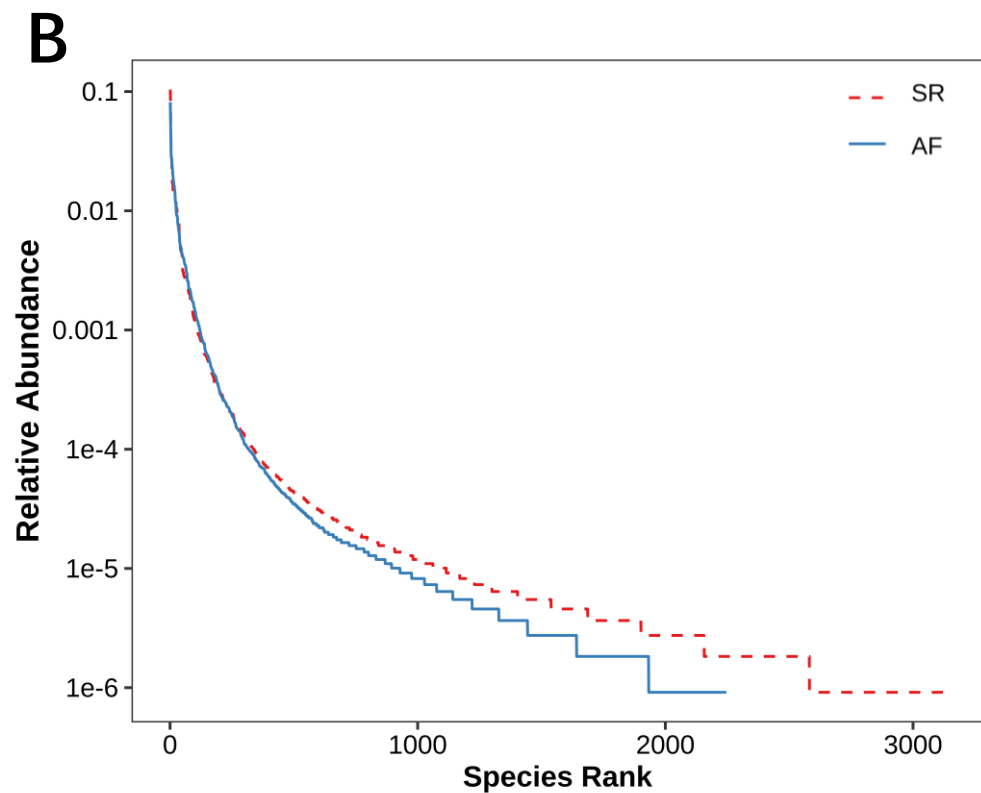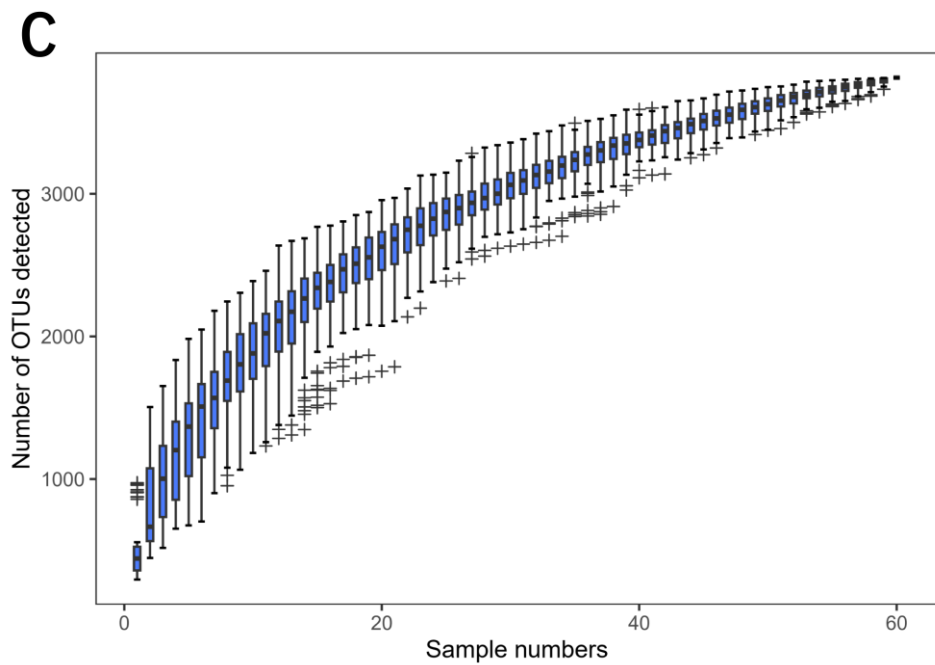

Supplement: Supplemental Information 3 — The gut microbiota richness in atrial fibrillation patients. (A) Rarefaction curves to show the adequate depth of the sequencing. (B) Rank abundance curve to show the relative species abundance of each sample from AF and SR patients. (C)Species accumulation boxplot to show that the number of samples is also adequate. [file peerj-11-16228-s003.pdf]

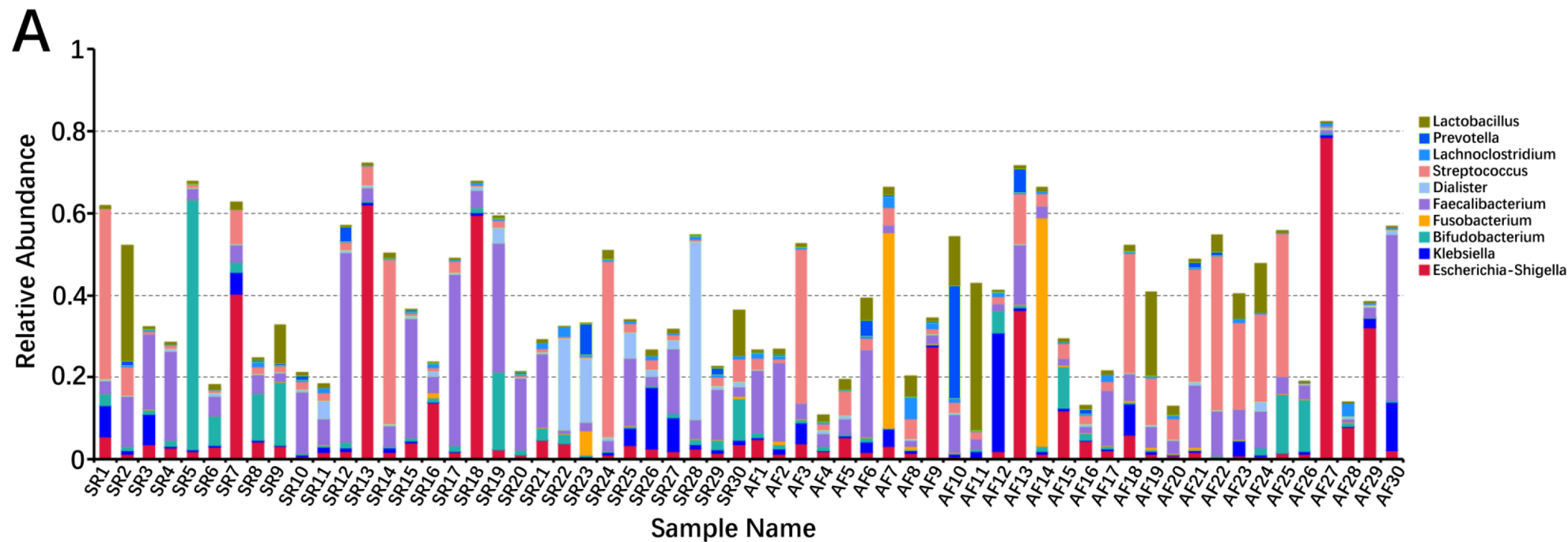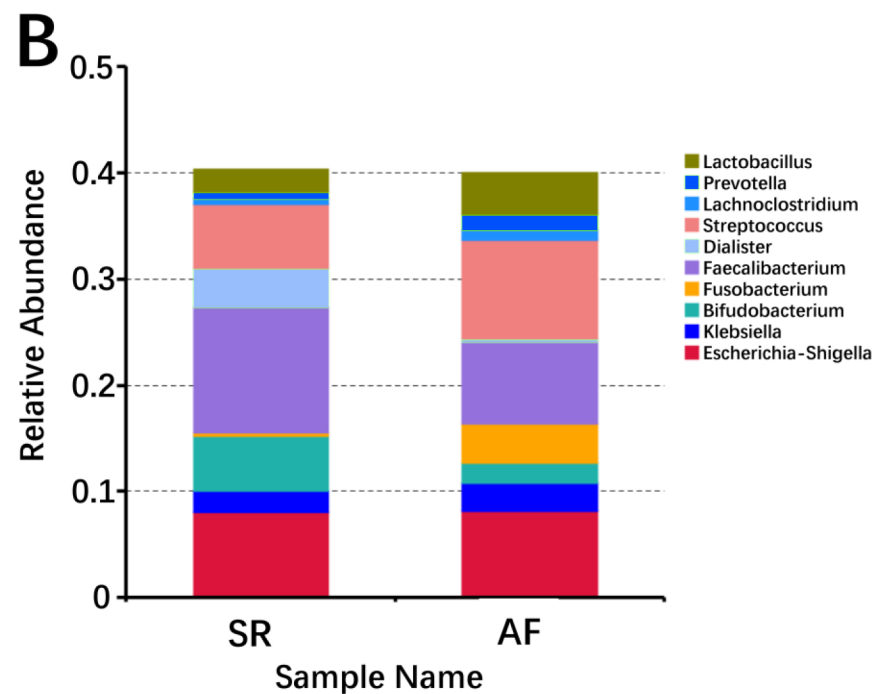

Supplement: Supplemental Information 4 — (A) Relative abundance of the top ten gut microbiota in each AF and SR patients. (B) Relative abundance of the top ten gut microbiota in AF and SR groups. The figure showed the species with top 10 gut microbiota in relative abundance. [file peerj-11-16228-s004.pdf]
